# The First Modified Multivisceral Transplantation in the Middle East: A Major Advance in Transplantation Surgery in Shiraz Transplant Center

**Published:** 2010-11-01

**Authors:** S. Nikeghalian, H. R. Davari, F. Kakaei, A. Shamsaeefar, B. Sanei, B. Geramizadeh, M. Moini, M. A. Sahmeddini, S. A. Malek-Hosseini

**Affiliations:** *Organ Transplant Research Center, Nemazi Hospital, Shiraz University of Medical Sciences, Shiraz, Iran.*

## INTRODUCTION

Multivisceral transplantation (MVTx) is the term used to describe concurrent transplantation of the stomach, pancreaticoduodenal complex, small intestine, and liver. Modified MVTx (MMVTx), the liver is not transplanted [1]. This technique was first described by Starzl, *et al*, [2] in dogs in 1960. The first patient who underwent this procedure was a 6-year-old girl with liver failure due to short bowl syndrome in 1983 [3]. With introduction of cyclosporine, survival beyond the immediate post-operative period became possible [3] so that the first patient who discharged from hospital after this procedure was reported in December 1989 [4]. In spite of development of new immunosuppressive protocols (especially the use of induction therapy with monoclonal antibodies and use of tacrolimus for preventing intestinal rejection), new surgical techniques, novel diagnostic instruments for graft monitoring, and better candidate selection, this procedure is rarely performed in the world [1,5,6]. Herein, we report on our first experience with MMVTx in a 30-year-old man with advanced desmoid tumor in Shiraz Transplant Center, Namazi Hospital, Shiraz, southern Iran. 

## CASE REPORT

**Figure 1:**
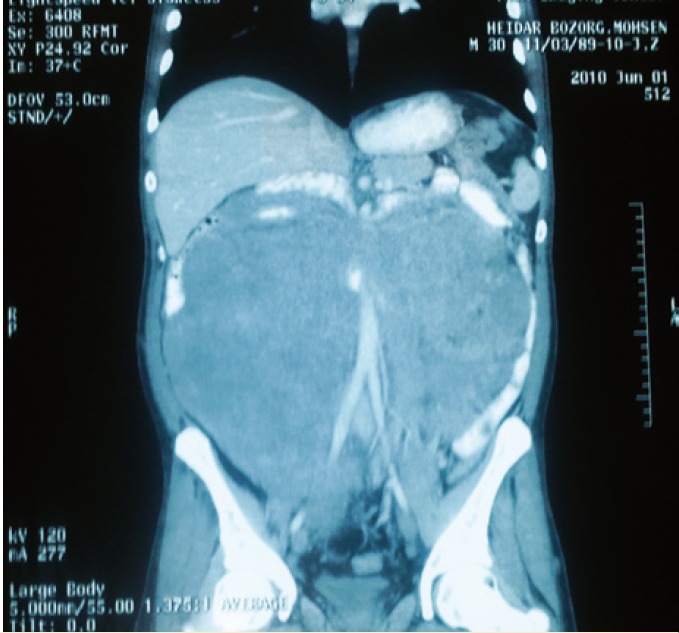
Preoperative abdominal computed tomography of the patient

In June 2009, a 30-year-old man was presented to the surgery clinic of a general hospital with painless right abdominal mass without any other problems. He underwent laparotomy where the surgeon found an inoperable mass that involved retroperineal area around ascending and transverse colon; then, he only performed an incisional biopsy and referred the patient to our center. The pathologic diagnosis was “desmoids tumor.” At presentation to our center, he developed transient hematochesia. Colonoscopy revealed multiple polyps of “familial adenomatous polyposis” type which involved the entire colon. Whole body spiral computed tomography (CT) demonstrated no signs of distant metastasis (Fig 1). The patient then underwent laparotomy for resection of the tumor and total colectomy with ileo-rectal anastomosis.

After the operation, he lost the follow-up, but 10 months later, in May 2010, he returned to our clinic in Namazi Hospital with a large abdominal mass and over 12 kg weight loss because of severe loss of appetite. Abdominal CT revealed recurrence of the tumor with involvement of all abdominal organs except for the left kidney and liver. No signs of distant metastasis were found in the imaging studies. After discussion about unresectability of his tumor with him and his family, we decided to perform an elective MMVTx for him.

Donor operation

**Figure 2:**
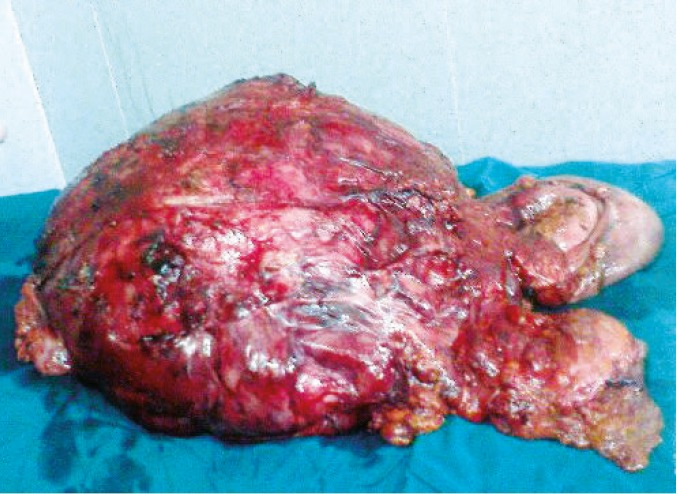
The excised tumor which completely encased other abdominal viscerae

Donor was a 28-year-old hemodynamically-stable head injured patient whose brain death was confirmed three hours before the operation. He had been admitted to intensive care unit (ICU) since 48 hours before his brain death was confirmed. He was only receiving 5 µg/kg/min dopamine as a vasoactive agent. His blood group was the same as the recipient. We used a long midline incision for exposing the heart and abdominal viscera. After complete dissection of abdominal organs including the arterial branches of celiac trunk and canulating the infra-renal aorta and inferior mesenteric vein, cold irrigation was started by University of Wisconsin (UW) solution. For removal of the liver, the hepatic artery was transected just after the origin of the gastro-duodenal artery and portal vein was transected just above the upper border of the pancreas. After removal of the liver and kidneys, the complex modified multivisceral graft (*en bloc* stomach, duodenum with pancreas and spleen and whole small intestine) was harvested using these steps: transecting of the esophago-gastric junction and terminal ileum by separate linear staplers; separating the splenic ligaments; and dissecting all retroperitoneal adhesions of the stomach, pancreaticoduodenal complex and small intestine. The arterial inflow of this complex graft was superior mesenteric and celiac arteries on a common patch of the aorta; the venous outflow of the graft was through the portal vein, immediately superior to upper border of the pancreas (Fig 2).

Recipient operation

The abdomen was opened with previous midline incision. A huge retroperitoneally originated mass was found in the abdominal cavity that completely encased the small bowel, duodenum, pancreas and left kidney of the recipient. No signs of metastasis were found. Portal vein, hepatic artery and the right kidney (including renal artery and vein and ureter), inferior vena cava and aorta were meticulously dissected free from the tumor. The superior and inferior mesenteric arteries and left renal artery and other minor branches of aorta were transligated. After finishing tumor dissection, gastro-esophageal junction, the ileo-rectal anastomosis site and common bile duct were transected, portal vein was clamped, and tumor was completely removed from the abdominal cavity. Hepatic artery remained intact to perfuse the liver during the rest of operation.

Implantation was done through the following steps. End-to-side anastomosis of the donor aortic patch to the anterior side of aorta; end-to-end anastomosis of the graft and native portal vein; after reperfusion of the organs, anastomosis of the native esophagus to graft stomach; graft terminal ileum to the native rectal remnant by endoluminal staplers; and, end-to-side anastomosis of native bile duct to a Roux-en-Y loop of the graft and constructing a protecting loop ileostomy, 45 cm proximal to the ileo-rectal anastomosis in the left side of abdomen. Pyloroplasty was performed for enhancing the gastric emptying. Abdominal cavity was drained externally by two Jackson-Pratt type drains. Abdominal wall was repaired in two layers without any problem. Total duration of operation was nine hours including four hours for tumor resection. The total cold ischemia time of the graft was six hours; the total warm ischemia time was 76 minutes.

Immunosuppression

Alemtuzumab (Campath^®^, Berlex Laboratories, Montville, NJ, USA) (after explanting the abdominal organs and a repeated dose on day four of the operation) and 1000 mg methylprednisolone (Solu-Medrol^®^, Pharmacia and Upjohn Co, Kalamazoo, MI, USA) (daily, for three days) were used for the induction of immunosuppression. Maintenance immunosuppression was started on day one by tacrolimus (Prograf^®^, Astellas Pharmaceuticals, Deerfield, IL, USA) that increased to 6 mg/day for a target serum level of 12 ng/mL. Oral prednisolone started on day four (50 mg/day) and gradually decreased to 20 mg/day during next three weeks.

Post-operative course 

The patient was extubated two hours after the operation. Total parenteral nutrition which was started pre-operatively, continued for the next two weeks with 35 kcal/kg/day. Prophylactic antibiotics (ceftazidime and ampicillin-sulbactam) and also amphotericin and ganciclovir were continued for two weeks. Drains were removed after 10 days when the lymphatic drainage was less than 100 mL/day. After day seven, gastric biopsy with upper gastro-intestinal endoscopy and direct biopsy of the ileostomy site was done three times per week for detecting acute rejections. Only one episode of acute cellular rejection was observed after three weeks which was treated by three-day methylprednisolone pulse therapy. The blood sugar of the patient was <140 mg/dL without any need for insulin therapy. Diet was started after seven days and the patient was discharged from hospital 28 days after the operation with oral medications (tacrolimus, prednisolone, omeprazole, ciprofloxacin, folic acid, and fluconazole).

Ten days later, he returned back to hospital because of severe abdominal pain and tenderness and underwent exploratory laparotomy. The cause of pain was the strangulation of the terminal ileum (the segment between the ileostomy and the ileo-rectal anastomosis) through an internal hernia. The ischemic segment was resected and the anastomoses were revised with a new ileostomy. He was discharged for the second time from the hospital seven days later, and now, after three months of the operation, he is on oral diet and in a good condition except for an episode of cytomegalovirus gastritis which is under treatment by valganciclovir (Valcyte^®^, Genentech, Roche Pharmaceuticals, San Francisco, CA, USA).

## References

[1] Tzakis AG, Kato T, Levi DM (2005). 100 Multivisceral Transplants at a Single Center. Ann Surg.

[2] Starzl TE, Kaupp HA Jr (1960). Mass homotransplantation of abdominal organs in dogs. Surg Forum.

[3] Starzl TE, Rowe MI, Todo S (1989). Transplantation of multiple abdominal viscera. JAMA.

[4] Margreiter R, Konigsrainer A, Schmid T (1992). Successful multivisceral transplantation. Transplant Proc.

[5] Todo S, Tzakis A, Abu-Elmagd K (1995). Abdominal multivisceral transplantation. Transplantation.

[6] Abu-Elmagd K, Reyes J, Bond G (2001). Clinical intestinal transplantation: a decade of experience at a single center. Ann Surg.

[7] Vianna RM, Mangus RS, Tector AJ (2008). Current status of small bowel and multivisceral transplantation. Adv Surg.

[8] Moon JI, Selvaggi G, Nishida S (2005). Intestinal transplantation for the treatment of neoplastic disease. J Surg Oncol.

[9] Grant D (1999). Intestinal transplantation. 1997 report of the international registry. Transplantation.

[10] Anthony T, Rodríguez-Bigas MA, Weber TK, Petrelli NJ (1996). Desmoid tumors. J Am Coll Surg.

[11] Chatzipetrou MA, Tzakis AG, Pinna AD (2001). Intestinal transplantation for the treatment of desmoid tumors associated with familial adenomatous polyposis. Surgery.

